# Spironolactone Protects against Diabetic Cardiomyopathy in Streptozotocin-Induced Diabetic Rats

**DOI:** 10.1155/2018/9232065

**Published:** 2018-10-14

**Authors:** Wenjuan Liu, Wei Gong, Min He, Yemei Liu, Yeping Yang, Meng Wang, Meng Wu, Shizhe Guo, Yifei Yu, Xuanchun Wang, Fei Sun, Yiming Li, Linuo Zhou, Shengmei Qin, Zhaoyun Zhang

**Affiliations:** ^1^Division of Endocrinology and Metabolism, Huashan Hospital, Fudan University, 12 Wulumuqi Road, Shanghai 200040, China; ^2^Institute of Endocrinology and Diabetology, Fudan University, 12 Wulumuqi Road, Shanghai 200040, China; ^3^Department of Endocrinology, The Second People's Hospital, 4 Duchun Road, Wuhu, Anhui 241001, China; ^4^Department of Endocrinology, The Second Affiliated Hospital, Soochow University, 1055 Sanxiang Rd, Suzhou, Jiangsu 215000, China; ^5^Department of Cardiology, Zhongshan Hospital, Fudan University, 180 Fenglin Road, Shanghai 200032, China

## Abstract

Spironolactone (SPR) has been shown to protect diabetic cardiomyopathy (DCM), but the specific mechanisms are not fully understood. Here, we determined the cardioprotective role of SPR in diabetic mice and further explored the potential mechanisms in both *in vivo* and *in vitro* models. Streptozotocin- (STZ-) induced diabetic rats were used as the in vivo model. After the onset of diabetes, rats were treated with either SPR (STZ + SPR) or saline (STZ + NS) for 12 weeks; nondiabetic rats were used as controls (NDCs). In vitro, H9C2 cells were exposed to aldosterone, with or without SPR. Cardiac structure was investigated with transmission electron microscopy and pathological examination; immunohistochemistry was performed to detect nitrotyrosine, collagen-1, TGF-*β*1, TNF-*α*, and F4/80 expression; and gene expression of markers for oxidative stress, inflammation, fibrosis, and energy metabolism was detected. Our results suggested that SPR attenuated mitochondrial morphological abnormalities and sarcoplasmic reticulum enlargement in diabetic rats. Compared to the STZ + NS group, cardiac oxidative stress, fibrosis, inflammation, and mitochondrial dysfunction were improved by SPR treatment. Our study showed that SPR had cardioprotective effects in diabetic rats by ameliorating mitochondrial dysfunction and reducing fibrosis, oxidative stress, and inflammation. This study, for the first time, indicates that SPR might be a potential treatment for DCM.

## 1. Introduction

Diabetic cardiomyopathy (DCM), which was first described by Rubler et al. in 1972 [1], is now used to refer to ventricular dysfunction in patients with diabetes that is out of proportion to their underlying vascular disease [2]. In 2014, it was estimated that the cumulative probability of death among patients with DCM was 18%, and the incidence of heart failure was 22% [3]. Thus, there is an urgent need to develop drugs that could prevent or reverse DCM.

It is well accepted that multiple pathogeneses are involved in the development of DCM, including mitochondrial dysfunction, impaired calcium handling, increased oxidative stress, endothelial dysfunction, and remodeling of the extracellular matrix [2]. More recently, an array of evidence showed that mitochondrial dysfunction may play a critical role in the development of DCM [4]. In diabetic conditions, mitochondrial dysfunction is related to decreased energy productivity [5], increased cellular oxidative stress [6, 7], impaired calcium handling [8], and impaired mitochondrial dynamics and biogenesis [9, 10].

The renin-angiotensin-aldosterone system (RAAS) plays an important role in the onset and progression of diabetes-induced cardiac dysfunction [11]. This system is composed of two parts: circulating RAAS (also known as the main part of this system) and local RAAS. Luik et al. reported that the activity of local RAAS in kidney was significantly increased in patients with uncomplicated type 1 diabetes, who were even normotensive and normoalbuminuric; however, the activity of circulating RAAS remained unchanged, or even decreased [12, 13]. Furthermore, hyperglycemia directly activated local RAAS in the heart [14]. The overactivation of RAAS results in the accumulation of aldosterone, which has been confirmed to aggravate myocardial oxidative stress and fibrosis [15]. Moreover, the activation of RAAS was shown to increase cardiac mitochondrial damage and subsequent oxidative stress in aging heart [16] and chronic heart failure (CHF) [17], while blockade of the RAAS could protect mitochondrial function of the skeletal muscle in type 2 diabetic mice [18].

Spironolactone (SPR), an antagonist of the aldosterone receptor, is widely used to treat chronic heart failure and edematous conditions. Clinical research verified that aldosterone receptor antagonists ameliorated left ventricular (LV) hypertrophy and improved LV structural remodeling [19–21]. Moreover, Miric et al. found that short-term SPR treatment reversed cardiac fibrosis and attenuated the increased diastolic stiffness in STZ-induced diabetic rats [22]. In addition, SPR was found to inhibit the production of proinflammatory factors in mononuclear cells and decrease oxidative stress in endothelial cells, which might mediate the protective effects in different heart diseases [23, 24]. In summary, all these clinical and basic researches show the potential utility of SPR in the treatment of DCM. However, the potential mechanism of the protective effects of SPR on DCM remains unclear. In a STZ-induced diabetic rat model, here we demonstrate that SPR protects diabetic cardiomyopathy mainly through reducing cardiac fibrosis, oxidative stress, and inflammation, as well as improving mitochondrial dysfunction.

## 2. Materials and Methods

### 2.1. Animal Study

All animal experiment protocols were approved by the Institutional Animal Care and Use Committee (IACUC) at Fudan University (animal protocol 20150524A225) and carried out in accordance with the guidelines of the institution. Five-week-old male Sprague-Dawley (SD) rats were purchased from SLRC Laboratory Animal (Shanghai, China). Diabetes was induced with a single dose of streptozotocin (STZ, 65 mg/kg in 0.1 mol/L citrate buffer solution, pH 4.5; Sigma, St. Louis, Missouri, USA) intraperitoneally injected as previously described [22] and confirmed when blood glucose levels reached 16.7 mmol/L 1 week after injection. All rats were housed in a specific pathogen-free animal vivarium and maintained on a 12 h light, 12 h dark cycle, with free access to standard rodent chow and water.

Diabetic SD rats were treated by gavage either with SPR (Xinyi Pharmaceutical Company, Shanghai, China; *n* = 8, 20 mg/kg per day, STZ + SPR group) or saline (*n* = 8, 0.1 mL/10 g body weight per day, STZ + NS group) for 12 weeks after the onset of diabetes. For the preparation of SPR solution, one water-insoluble tablet of SPR was resuspended in 10 mL saline. Before each gavage, SPR solution was vortexed to make a homogenous suspension. Eight nondiabetic control (NDC) rats received the same saline gavage (*n* = 8, 0.1 mL/10 g body weight per day).

Body weight and random blood glucose levels were checked weekly. Blood pressure was measured by tail-cuff plethysmography monthly (BP-98A, Softron Beijing Incorporated, Beijing, China). After 12 weeks [25], all animals were sacrificed and heart tissues were isolated for further analysis.

### 2.2. Cell Culture

Rat cardiomyocyte-like H9C2 cells were cultured with Dulbecco's modified Eagle's medium (DMEM; Invitrogen, Waltham, Massachusetts, USA) supplemented with 10% fetal bovine serum (FBS; Invitrogen, USA) and 1% penicillin/streptomycin (Invitrogen, USA) in a humidified incubator (5% CO_2_) at 37°C. At 80% confluence, H9C2 cells were treated with aldosterone (10^−7^ mol/L) [24, 26] and SPR (10^−7^ mol/L) [22]. After 72 h, cells were collected for further analysis.

### 2.3. Histologic Evaluation

Left ventricular myocardia were fixed in 4% paraformaldehyde overnight. Tissues were embedded in paraffin, stained with hematoxylin and eosin (H&E) or PAS or with Masson reagent as described before [27], and examined under an optical microscope (Olympus, Richmond Hill, ON, Canada). The cross-sectional area of single myocytes was measured with ImageJ software [28]. The outline of 100–200 cardiomyocytes was traced in each group. For the quantification of PAS and Masson staining, each section was captured for at least 10 images. ImageJ was used to do the analysis, and the result was expressed as the percentage of PAS (or Masson) staining positive area in the total area of the cross section [29, 30].

Left ventricular samples for electron microscopy were cut into approximately 1 mm^3^ pieces and fixed in 10% glutaraldehyde overnight. Tissues were then fixed in 1% osmium tetroxide diluted with 1% K_4_Fe(CN)_6_, dehydrated through graded concentrations of ethanol and propylene oxide, and then embedded in Epon 812 as previously described [31]. Ultrathin sections were cut from blocks and mounted on copper grids. The grids were then counterstained with lead citrate and uranyl acetate, observed with a transmission electron microscope (FEI Tecnai G2 Spirit, Hillsboro, Oregon, USA), and photographed.

### 2.4. Immunohistochemistry Analysis

Left ventricles were fixed with 4% paraformaldehyde overnight and then embedded in paraffin. Expression of nitrotyrosine (2459610, Millipore, Billerica, MA), collagen 1 (ab34710, Abcam), TGF-*β*1 (sc-146, Santa Cruz Biotechnology Inc.), TNF-*α* (ab6671, Abcam), and F4/80 (70076, Cell Signaling Technology) on tissue sections (5 mm) was examined by immunohistochemical analysis as previously described [32]. The dilution factor was 1 : 100, 1 : 200, 1 : 100, 1 : 250, and 1 : 100, respectively. For IHC analysis, each section was captured in at least 10 images and all images were quantified using Image-Pro Plus 6.0 software (Media Cybernetics, Silver Spring, Maryland, USA). For nitrotyrosine, collagen 1, TGF-*β*1, and TNF-*α*, data were expressed as the ratio of integrated optical density (IOD) to area. Percentages of positive stains for F4/80 within the fields were averaged for each mouse and then for each group as described before [33].

### 2.5. RNA Extraction and Real-Time Polymerase Chain Reaction (Real-Time PCR)

RNA was extracted from the left ventricles or cultured cells using TRIzol reagent (Life Technologies, Waltham, Massachusetts, USA). RNA concentration was determined using a spectrophotometer (NanoDrop 2000c, Thermo Scientific, Waltham, Massachusetts, USA) by absorbance at 260 nm. Then, 2 *μ*g of total RNA was converted to cDNA according to the manufacturer's protocol (G490, Abm, Canada). cDNA was analyzed for the expression of target genes with the ABI 7500 Sequence Detection System (Applied Biosystems, Waltham, Massachusetts, USA) using the following conditions: 94°C, 5 min; 94°C, 30 seconds, 55°C, 30 seconds, 72°C, 1 minute 30 seconds, 40 cycles; 72°C, 10 min. Each sample was run in triplicate to permit precise quantification of each gene normalized to the GAPDH gene. The primers used in this study are shown in Table 1.

### 2.6. Protein Extraction and Western Blot Analysis

The left ventricles were homogenized in an ice-cold RIPA lysis buffer (Thermo Scientific, Waltham, MA) with a protease inhibitor cocktail (Roche Applied Science, Mannheim, Germany). These samples were loaded in 10% sodium dodecyl sulfate-polyacrylamide gel electrophoresis and transferred to polyvinylidene difluoride membranes (Millipore). The membranes were then incubated with rabbit anti-collagen 1 (1 : 5000, Abcam, Cambridge, MA, USA), rabbit anti-TGF-*β*1 (1 : 1000, Abcam), or rabbit anti-tubulin (1 : 5000, Millipore) antibodies overnight. After that, membranes were washed and incubated with goat anti-rabbit HRP-coupled secondary antibodies (1: 10,000). The results were visualized by an enhanced chemiluminescence detection system (PerkinElmer, Boston, MA, USA).

### 2.7. Quantification of Mitochondrial DNA (mtDNA) Content by Quantitative Real-Time PCR

Total genomic DNA was precipitated from left ventricles as previously described [34]. Approximately 15 mg tissues were homogenized and digested with proteinase K at 55°C overnight in a lysis buffer for DNA extraction by a conventional phenol-chloroform method. The DNA concentrations were measured using a NanoDrop Spectrophotometer ND-1000 (Thermo Scientific, Waltham, MA). The results were calculated with the difference in the threshold cycle values for mtDNA and nuclear specific amplification by quantitative real-time PCR. The data are presented as mtDNA-specific 12S ribosomal RNA normalized to the nuclear specific gene *β*-actin. The primers for mtDNA (Era-like 12S mitochondrial rRNA chaperone 1, Eral1) and nuclear DNA (*β*-actin) are shown in Table 2.

### 2.8. Measurement of Cellular ROS Levels

Cellular ROS levels were determined using a 2′,7′-dichlorofluorescein diacetate (DCF-DA) probe according to the manufacturer's instructions (D399, Invitrogen, MA, USA 02451). Briefly, 4 × 10^4^ H9C2 cells were seeded in 96-well plates and grown overnight. After treatment with different conditions for 72 hours, cells were washed once with PBS and loaded with 10 *μ*M H2DCFDA. After incubation at 37°C for 30 minutes, the dye was removed and cells were washed once with PBS and scanned with a plate reader at 490/526 nm emission (Molecular Devices, Gemini XS, Sunnyvale, CA, USA), and background fluorescence was subtracted from each corresponding well. Results were expressed as the ratio of fluorescence intensity compared with untreated controls.

### 2.9. Statistical Analysis

All statistical analyses were performed using SPSS for Mac Ver. 20.0 (SPSS Inc., Chicago, IL, USA). Data are expressed as mean ± SEM. The significance among different groups was evaluated using one-way ANOVA followed by the Tukey test. All graphs were made using PRISM program (GraphPad, San Diego, California, USA). Differences were considered statistically significant for *P* values <0.05.

## 3. Results

### 3.1. Animal Characteristics

Rats of both STZ + NS and STZ + SPR groups developed robust, sustained, and equivalent hyperglycemia, reduced body weight, increased heart weight, and decreased heart weight/body weight ratio compared to those in the NDC group (Table 3). Systolic and diastolic blood pressures were not significantly different in diabetic rats compared with those in the NDC group (Table 3). Blood glucose, heart weight, and heart weight/body weight ratio were not different between the STZ + NS group and the STZ + SPR group (Table 3).

### 3.2. Cardiac Ultrastructure Was Preserved in SPR-Treated Diabetic Rats

**Figure 1:**
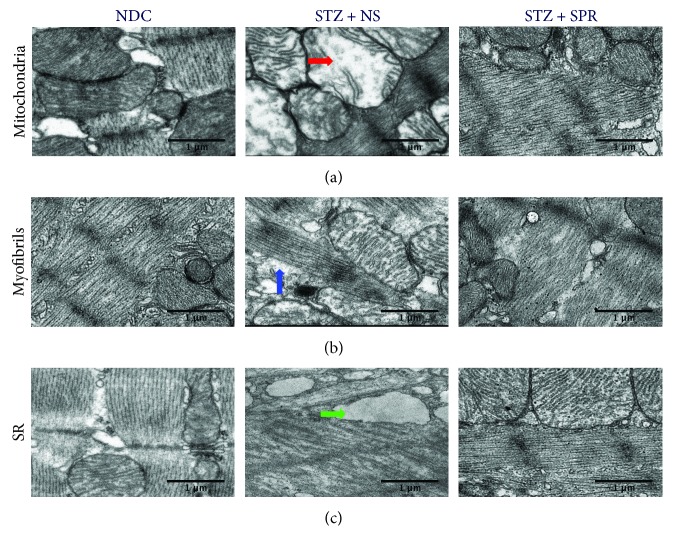
Cardiac ultramicrostructure was preserved in SPR-treated rats. (a–c) Representative electron micrographs for mitochondria, myofibrils, and sarcoplasmic reticulum, respectively. In the NDC group, normal ultrastructure was observed in the heart tissue. Loss of cristae, irregular arrangement of myofibrils, and enlarged sarcoplasmic reticula were observed in the STZ + NS group. Pathological changes in the STZ + SPR group were significantly reduced compared to those of the diabetic group. Red, blue, and green arrows indicate mitochondria, myofibrils, and sarcoplasmic reticulum, respectively.

As shown in Figure 1(a), compared with the NDC, ballooning and loss of cristae (red arrow) were seen in mitochondria of myocytes from the left ventricular myocardium in diabetic rats, whereas normal mitochondria were observed in the myocardium of SPR-treated diabetic rats. In Figure 1(b), compared with the NDC group, the STZ + NS group displayed disordered sarcomeres, shown by the breakdown and irregular arrangement of myofibrils (blue arrow). Adjacent sarcomeres displayed Z band misalignments, disruptions, irregularities, and breakdowns. However, these abnormalities were ameliorated by SPR treatment (Figure 1(b)). Compared with the NDC group, the sarcoplasmic reticulum in the STZ + NS group rats was enlarged (Figure 1(c), green arrow), which indicated an abnormality in calcium metabolism. However, these pathological changes were also protected in STZ + SPR group rats (Figure 1(c)).

### 3.3. SPR Abolished Myocardial Oxidative Stress in Diabetic Rats

**Figure 2:**
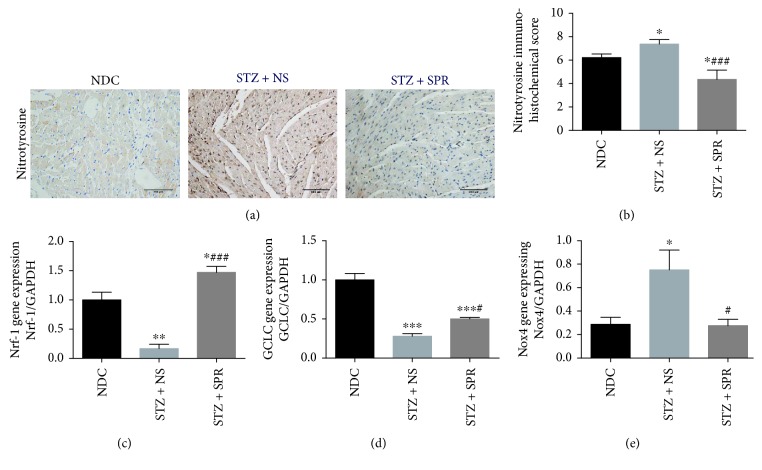
SPR abolished myocardial oxidative stress in diabetic rats. (a) Representative immunohistochemical (IHC) micrographs of myocardium tissue from NDC, STZ + NS, and STZ + SPR groups stained with nitrotyrosine. Magnification 20×. (b) Immunohistochemical scores for the IHC staining of myocardium sections. (c, d) Gene expression of Nrf-1 and GCLC for these three groups. *N* = 8, data are shown as means ± SEM. ^∗^*P* < 0.05, ^∗∗^*P* < 0.01, and ^∗∗∗^*P* < 0.001 vs. NDC group; ^#^*P* < 0.05 and ^###^*P* < 0.001 vs. STZ + NS group.

Myocardial oxidative stress was evaluated by IHC staining of nitrotyrosine, a marker of oxidative stress, as well as by gene expression of nuclear respiratory factor-1 (Nrf-1), glutamate-cysteine ligase catalytic subunit (GCLC), and nicotinamide adenine dinucleotide phosphate oxidase 4 (Nox4). Compared to NDC rats, there was a significant accumulation of nitrotyrosine in the hearts of STZ + NS group rats, which was reduced by SPR treatment (Figures 2(a) and 2(b)). Previous studies have shown the involvement of Nrf-1, GCLC, and Nox4 in oxidative stress [35–37]. Consistently, we found that the mRNA levels of Nrf-1 and GCLC in the STZ + NS group were significantly decreased compared to those of the NDC group (Figures 2(c) and 2(d)), while the mRNA expression of Nox4 was increased (Figure 2(e)). However, SPR increased the expression of Nrf-1 and GCLC while it decreased the expression of Nox4 (Figures 2(c)–2(e)). These results suggested that SPR attenuated myocardial oxidative stress by promoting the expression of several antioxidative components, while suppressing the expression of oxidative components.

### 3.4. SPR Protected against Myocardial Hypertrophy and Fibrosis in DCM

**Figure 3:**
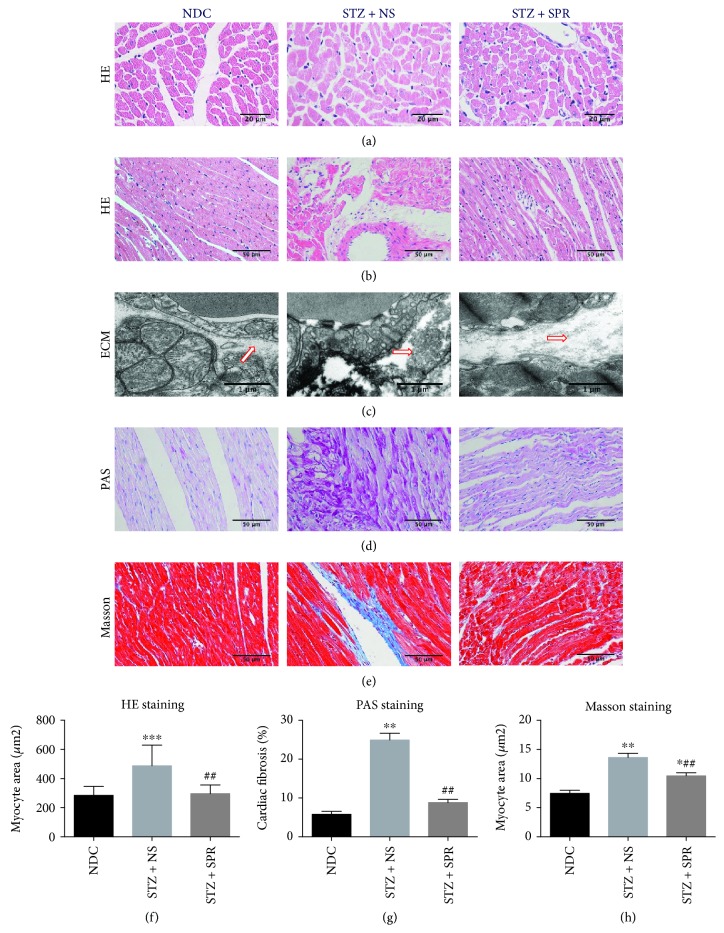
SPR protected against myocardial hypertrophy and fibrosis in DCM. (a) Representative images of cardiac tissue stained with HE (transsection) for NDC, STZ + NS, and STZ + SPR groups (original magnification 40×). (b) Representative images of cardiac tissue stained with HE (longisection) for NDC, STZ + NS, and STZ + SPR groups (original magnification 20×). (c) Representative electron micrographs for the extracellular matrix from NDC, STZ + NS, and STZ + SPR groups. Compared to the NDC group, the deposition of the extracellular matrix (ECM) tended to be increased in the STZ + NS group, while SPR treatment reduced this pathological change. Blue arrows indicate ECM. (d, e) Representative images of cardiac tissue stained with PAS and Masson for the NDC, STZ + NS, and STZ + SPR groups are presented (original magnification 20×). (f, e) Quantification results summarizing the cross-sectional diameter of myocytes within transverse cardiac sections. (g, h) Quantification results of the PAS and Masson staining positive area in cardiac sections of rats treated under the conditions indicated, respectively. *N* = 8 for each group, data are shown as means ± SEM. ^∗^*P* < 0.05, ^∗∗^*P* < 0.01, and ^∗∗∗^*P* < 0.001 vs. NDC group; ^##^*P* < 0.01 vs. STZ + NS group.

H&E, PAS, and Masson staining were used to evaluate the pathological changes in the heart. H&E staining revealed that in the STZ + NS group, cardiomyocytes presented significant hypertrophy compared to the NDC group, which was largely reversed by SPR treatment (Figures 3(a) and 3(f)). Moreover, compared with the NDC group, the arrangement of the cardiac fibers was disrupted and the intercellular borders were obscure in the STZ + NS group, while SPR treatment significantly improved these abnormalities (Figure 3(b)).

Electron microscope examination showed that the deposition of the extracellular matrix (ECM) was increased in the STZ + NS group compared with that in the NDC group, while this was significantly abolished by SPR treatment (Figure 3(c)). In addition, we measured the extracellular matrix and collagen accumulation by PAS (Figures 3(d) and 3(g)) and Masson staining (Figures 3(e) and 3(h)), respectively. Our results demonstrated that there was fibrosis in the STZ + NS group, which further supported the existence of DCM in this STZ-induced rodent model. More importantly, SPR treatment ameliorated these pathological abnormalities in the diabetic hearts (Figures 3(d), 3(e), 3(g), and 3(h)).

**Figure 4:**
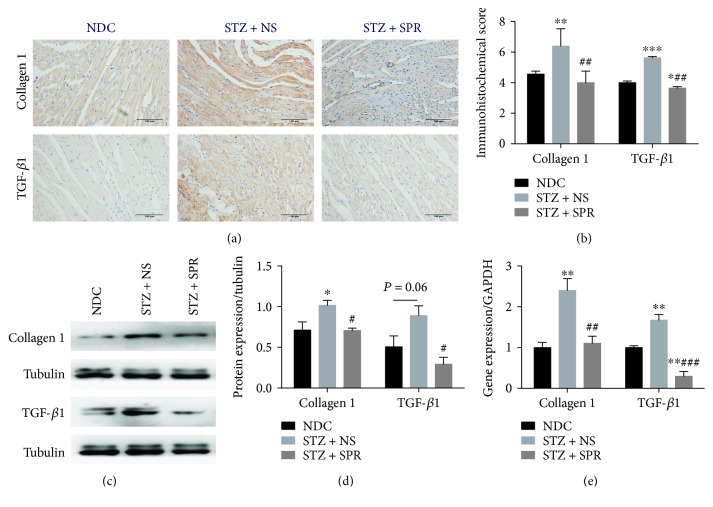
SPR protected against myocardial hypertrophy and fibrosis in DCM. (a) Representative immunohistochemical micrographs of cardiac tissue stained with collagen 1 and TGF-*β*1 (original magnification 20×). (b) The IHC scores of heart sections for collagen 1 and TGF-*β*1 IHC staining. (c) The protein expression of collagen 1 and TGF-*β*1 examined by Western blot. (d) The quantification data of Western blot for collagen 1 and TGF-*β*1. (e) Gene expression of collagen 1 and TGF-*β*1 for these 3 groups. *N* = 8, data are shown as means ± SEM. ^∗^*P* < 0.05, ^∗∗^*P* < 0.01, and ^∗∗∗^*P* < 0.001 vs. NDC group; ^#^*P* < 0.05, ^##^*P* < 0.01, and ^###^*P* < 0.001 vs. STZ + NS group.

We further performed IHC, Western blotting, and RT-PCR to test the expressions of collagen 1 and transforming growth factor-*β*1 (TGF-*β*1) (Figure 4(a)). Compared to the NDC group, the STZ + NS group showed significant increased accumulation of these two fibrotic components, which were reduced in the STZ + SPR group (Figures 4(a) and 4(b)). Furthermore, we also found that the mRNA and protein expression of collagen 1 and TGF-*β*1 was also substantially decreased by SPR (Figures 4(c)–4(e)).

### 3.5. SPR Reduced Cardiac Inflammation in DCM

**Figure 5:**
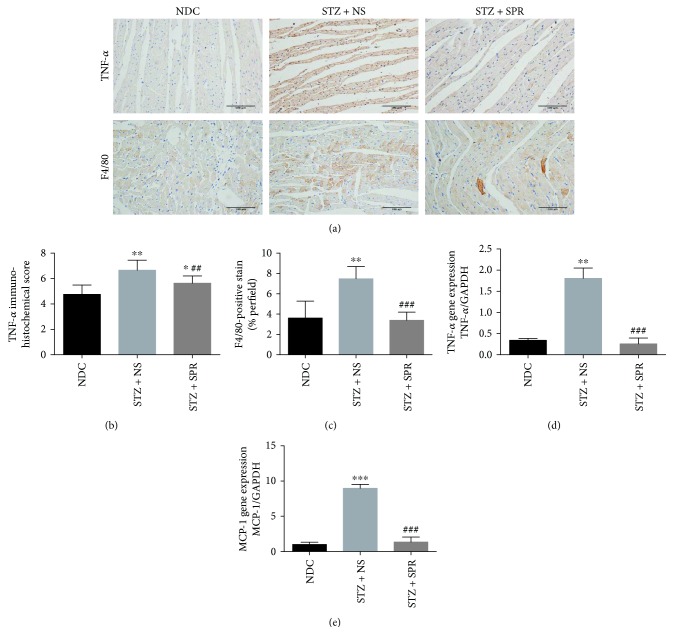
SPR reduced cardiac inflammation in DCM. (a) Representative immunohistochemical micrographs of cardiac tissue stained with TNF-*α* and F4/80 for NDC, STZ + NS, and STZ + SPR groups, respectively (original magnification 20×). (b) The IHC scores of heart sections for TNF-*α* IHC staining. (c) The percentage of positive staining per section for F4/80 IHC staining. (d, e) Gene expression of TNF-*α* and MCP-1 for these three groups, respectively. *N* = 8, data are shown as means ± SEM. ^∗^*P*<0.05, ^∗∗^*P*<0.01, and ^∗∗∗^*P*<0.001 vs. NDC group; ^##^*P* < 0.01 and ^###^*P* < 0.001 vs. STZ + NS group.

It is well recognized that oxidative stress promotes the expression of key components involved in the inflammatory signaling pathways, which might further contribute to cardiac damage. Moreover, the increased aldosterone levels in diabetic rodents could further lead to immune activation, e.g., through the NLRP3 inflammasome and T-regulatory cells, which in turn might further aggregate oxidative stress [38, 39]. Therefore, we assessed myocardial inflammation by detecting the protein (Figures 5(a) and 5(b)**)** and mRNA expression (Figure 5(d)) of TNF-*α*, the infiltration of cardiac macrophages (F4/80-positive cells, Figures 5(a) and 5(c)), and the mRNA expression of MCP-1 (Figure 5(e)). The results showed a significant increase in the expression of these inflammatory markers in diabetic rats compared with NDC rats, while it was ameliorated by SPR.

### 3.6. SPR Restored the Mitochondrial Contents and Improved Myocardial Energy Metabolism in Diabetic Rats

**Figure 6:**
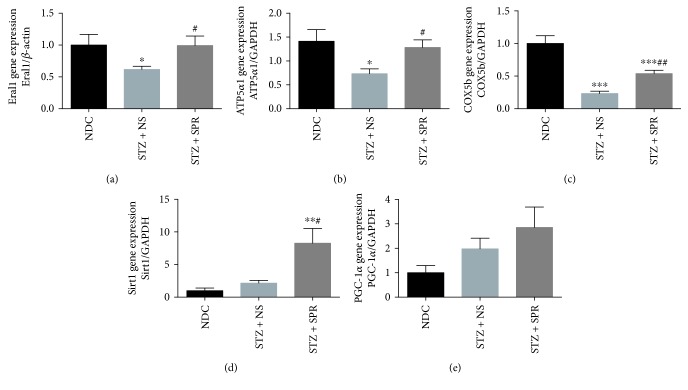
SPR restored the mitochondria contents and improved myocardial energy metabolism in diabetic rats. (a–e) Gene expression of Eral1, ATP5*α*1, COX5b, Sirt1, and PGC-1*α* for NDC, STZ + NS, and STZ + SPR groups. *N* = 8, data are shown as means ± SEM. ^∗^*P* < 0.05, ^∗∗^*P* < 0.01, and ^∗∗∗^*P* < 0.001 vs. NDC group; ^#^*P* < 0.05 and ^##^*P* < 0.01 vs. STZ + NS group.

As shown in Figure 1(a), SPR protected the ultrastructure of mitochondria, so we further investigated the underlying mechanism. Mitochondrial contents were evaluated by examining the DNA expression levels of Era-like 12S mitochondrial rRNA chaperone 1 (Eral1) (Figure 6(a)), which is a GTPase that localizes to the mitochondria and associates with mitoribosomal proteins including the 12S rRNA [40]. Our result showed that compared to NDC rats, the mitochondrial number in the cytoplasm of diabetic cardiomyocytes was decreased, while SPR treatment restored the mitochondrial contents (Figure 6(a)). Next, we examined the mRNA expression of genes involved in energy metabolism, including ATP synthase 5a (ATP5*α*1), cytochrome oxidase 5b (COX5b), NAD-dependent deacetylase sirtuin-1 (Sirt1), and peroxisome proliferator-activated receptor gamma coactivation factor 1*α* (PGC-1*α*). It showed that compared to the NDC group, the myocardial expression of ATP5*α*1 and COX5b was decreased in the STZ + NS group which was increased by SPR treatment (Figures 6(b) and 6(c)). However, in STZ-induced diabetic rats, SPR significantly increased the expression of and Sirt1 (Figure 6(d), respectively), while it had no effect on PGC-1*α* expression (Figure 6(e)).

### 3.7. SPR Decreased Oxidative Stress Induced by Aldosterone in a Myocardioblast Cell Line

**Figure 7:**
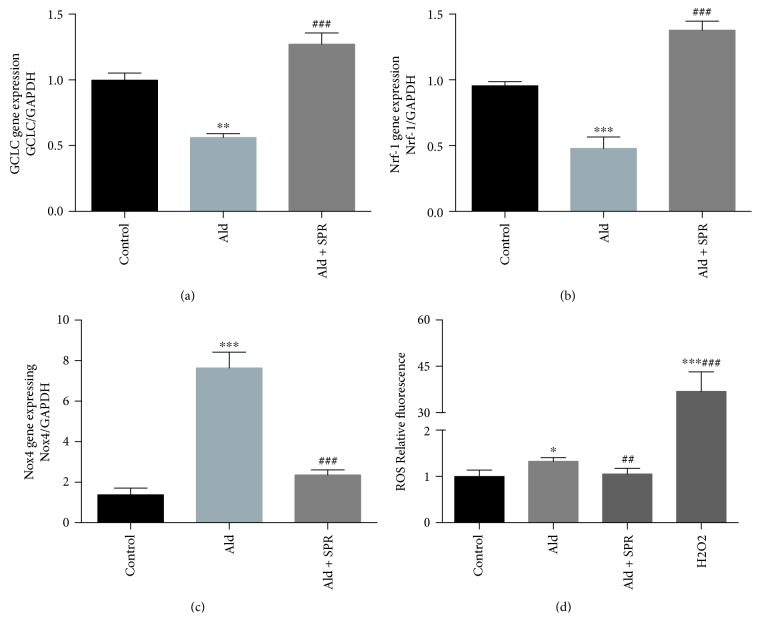
SPR aborted the aggregated oxidative stress induced by aldosterone in a myocardioblast cell line. (a–c) Gene expression of GCLC, NRF-1, and Nox4 in H9C2 cells treated with aldosterone (10^−7^ mmol/L), with or without SPR (10^−7^ mol/L), for 72 h. (d) Cellular ROS levels after treatment with aldosterone, with or without SPR. *N* = 3, data are shown as mean ± SEM. ^∗^*P* < 0.05, ^∗∗^*P* < 0.01, and ^∗∗∗^*P* < 0.001 vs. control group; ^##^*P* < 0.01 and ^###^*P* < 0.001 vs. aldosterone-treated group.

To further explore the mechanism underlying the protective effects of SPR on DCM, H9C2 cells were treated with aldosterone (10^−7^ mmol/L), with or without SPR (10^−7^ mol/L), for 72 h. Aldosterone significantly decreased the expression of GCLC and Nrf-1 compared with the untreated control (Figures 7(a) and 7(b)), while it increased Nox4 expression (Figure 7(c)). These changes were all further abrogated by SPR treatment (Figure 7(a)–7(c)). We then assessed cellular ROS levels. As shown in Figure 7(d), aldosterone significantly increased cellular ROS levels compared with control cells, while SPR reversed this change.

## 4. Discussion

Diabetic cardiomyopathy (DCM) is mediated by multiple disordered metabolic statuses and signaling pathways, as previously reported [41–43]. Previous studies have shown that the overactivation of RAAS, mainly through aldosterone and angiotensin II, resulted in cardiac insulin resistance and a cascade of abnormalities [44]. A number of clinical studies have demonstrated that the administration of aldosterone antagonists improved left ventricular function in patients with CHF, hypertensive cardiomyopathy, metabolic syndrome, and diabetic vascular dysfunction [45–49]. However, the beneficial effects of aldosterone antagonism on DCM had not been fully evaluated, and the underlying mechanism is still unknown. In this study, we found that SPR had cardiac-protective effects in STZ-induced diabetic rats. This was exemplified by reduced myocardial oxidative stress, inflammation, and fibrosis, as well as improved pathological structures and normalized cardiac mitochondrial functions. Thus, SPR might be an effective medication for protecting against DCM.

Normal cardiac function is energetically demanding, relying on well-coupled mitochondria to generate adenosine triphosphate (ATP). The dysfunction of mitochondria in DCM results in the deficiency of cellular energy supply, which causes further accumulation of ROS [50–52]. Our study showed, for the first time, that SPR restored the mitochondrial contents and ameliorated the dysfunction of myocardial energy metabolism by elevating the gene expression of ATP5*α*1 and COX5b (both of which are key components of the electron transport chain). In our diabetic rodent model, the expression of ATP5*α*1 was significantly decreased in diabetic animals compared to nondiabetic rats, which was consistent with previous studies in mice [53], while SPR treatment reversed this change. We observed that there was no difference between SPR-treated diabetic rats and saline-treated diabetic rats in blood pressure and blood glucose levels, which indicated that the protective effects of SPR on DCM were independent of lowering glucose or blood pressure.

Apart from ATP production [7, 54], mitochondria are also the main organelles involved in ROS production. Recent studies showed that both type 1 and type 2 diabetic hearts had increased oxidative stress [41–43, 51, 55]. It is accepted that the systemic administration of aldosterone increases the formation of ROS in macrophages, heart, vasculature, and kidneys [56–59]. These effects are partially due to increased NADPH oxidase activity, decreased glucose-6-phosphate dehydrogenase (G6PD) expression, and/or activation of the nuclear factor kappa-light-chain-enhancer of activated B cell (NF-*κ*B) signaling pathway [57, 60]. Conversely, the administration of anti-aldosterone drugs attenuates oxidative stress in the heart, vasculature, and kidneys [61, 62]. However, it remained unclear whether SPR could reduce cardiac oxidative stress under diabetic conditions.

In this study, we investigated the accumulation of nitrotyrosine, an index for oxidative stress, and found that it was increased in hearts from STZ-induced diabetic rats. Furthermore, we also investigated the gene expression of Nrf-1, GCLC, and Nox4. Nrf-1, a transcription factor, promotes the expression of some key genes involved in antioxidative stress, cellular growth, and mitochondrial DNA transcription and replication [36]. Glutathione (GSH), an important cellular antioxidant, is synthesized from L-glutamate and cysteine via the enzymes glutamate cysteine ligase (GCL) and glutathione synthetase (GSS). GCL, a heterodimeric enzyme composed of a catalytic (GCLC) and a modulatory (GCLM) subunit, is the rate-limiting enzyme in this process [35]. Our results showed that in diabetic heart, Nrf-1 and GCLC were decreased, which was upregulated with SPR treatment. Nox4 is a major enzyme for the production of superoxide (O^2−^) [37]. We showed that the Nox4 expression in the heart of diabetic rats was upregulated while SPR treatment decreased the expression. Interestingly, a recent study showed that Nox4, expressed in both cardiomyocyte and endothelial cells, mediated the protection against hemodynamic overload-induced cardiac remodeling [63]. The discrepancy between this research and ours may be due to different rodent models. To our knowledge, this is the first study to report that SPR regulates the expression of key genes involved in the oxidative and antioxidative stress systems. Further studies are needed to explore the regulatory mechanisms.

Since the first report about the effect of increased plasma aldosterone on collagen accumulation in the myocardium [64], several studies have demonstrated that aldosterone induces cardiac remodeling and fibrosis [65, 66]. It was shown that aldosterone stimulated the expression of TGF-*β*, adhesion molecules (including intercellular adhesion molecule 1 (ICAM-1) and vascular cell adhesion protein 1 (VCAM-1)), growth factors, and metallothioneins [67]. Indeed, in patients with mildly dilated cardiomyopathy, hypertensive cardiomyopathy, or metabolic syndrome, the administration of spironolactone improved left ventricular diastolic function and led to regression of myocardial fibrosis [45–47]. More recently, a study showed that in nonhypertensive Zucker diabetic fatty rats, eplerenone attenuated cardiac steatosis and apoptosis, with a subsequent decrease in cardiac remodeling and diastolic dysfunction [68]. However, there has been no evidence showing the protective effects of SPR on cardiac fibrosis and inflammation in type 1 diabetic models. Hence, we examined the ultrastructure of myocardial tissue, pathological changes, and the gene and protein expression of certain markers for fibrosis (collagen 1 and TGF-*β*1) and inflammation (MCP-1 and TNF-*α*). Our results showed that SPR significantly reduced the cardiac fibrosis and inflammation in STZ-induced diabetic rats.

Although we measured the pathological changes and found the existence of cardiomyocyte hypertrophy and extensive deposition of the extracellular matrix and collagen in the myocardial interstitium, the in vivo cardiac function examination was lacking. Further in vivo studies are warranted to directly examine the effects of SPR on the cardiac function in this model.

## 5. Conclusion

In summary, we found that exogenous SPR had cardiac-protective effects in STZ-induced diabetic rats. This was exemplified by reduced myocardial oxidative stress, inflammation, and fibrosis, as well as improved pathological structures and normalized cardiac energy metabolism. Thus, SPR might be an effective medication to protect against DCM.

## Figures and Tables

**Table 1:** Sequences of primers used for real-time PCR.

	Gene access number	Forward	Reverse
Sirt-1	309757	5′-TGATTGGCACCGATCCTCG-3′	5′-TGGATGCTCTCATCAGGACAG-3′
PGC-1*α*	83516	5′-AAGTGGTGTAGCGACCAATCG-3′	5′-AATGAGGGCAATCCGTCTTCA-3′
ATP5*α*1	65262	5′-GAGACTGGGCGTGTGTTAAG-3′	5′-CTCCTCTGCTTGAACATTCCTC-3′
COX-5b	94194	5′-ACCCTAATCTAGTCCCGTCC-3′	5′-CAGCCAAAACCAGATGACAG-3′
TGF-*β*1	59086	5′-CCTGCAAGACCATCGACATG-3′	5′-TGTTGTACAAAGCGAGCACC-3′
Collagen 1	29393	5′-TCAAGATGGTGGCCGTTACT-3′	5′-CATCTTGAGGTCACGGCATG-3′
TNF-*α*	24835	5′-TCATCCGTTCTCTACCCAGC-3′	5′-TACTTCAGCGTCTCGTGTGT-3′
MCP-1	24770	5′-ACCAGCCAACTCTCACTGAA-3′	5′-GCCAGTGAATGAGTAGCAGC-3′
Nrf-1	312195	5′-GAGTGACCCAAACCGAACA-3′	5′-GGAGTTGAGTATGTCCGAGT-3′
GCLC	25283	5′-GATGATAGAACACGGGAGG-3′	5′-CATTGGTCGGAACTCTACTC-3′
Nox4	85431	5′-TGTCTGCATGGTGGTGGTAT-3′	5′-CTTCAACAAGCCACCCGAAA-3′
GAPDH	24383	5′-CTGGAGAAACCTGCCAAGTATGAT-3′	5′-TTCTTACTCCTTGGAGGCCATGTA-3′

Sirt-1: NAD-dependent deacetylase sirtuin-1; PGC-1*α*: peroxisome proliferator-activated receptor gamma coactivation factor 1*α*; ATP5*α*1: ATP synthase 5a; COX-5b: cytochrome oxidase 5b; TGF-*β*: transforming growth factor *β*; TNF-*α*: tumor necrosis factor *α*; MCP-1: monocyte chemoattractant protein 1; Nrf-1: nuclear respiratory factor 1; GCLC: glutamate cysteine ligase catalytic subunit; Nox4: NADPH oxidase 4; GAPDH: glyceraldehyde 3-phosphate dehydrogenase.

**Table 2:** Sequences of primers used for PCR.

	Gene access number	Forward	Reverse
Eral1	363646	5′-TGGTGCCCAAAGAGTCTCAT-3′	5′-TCCTGTAGACCCCTCTGACA-3′
*β*-Actin	81822	5′-CCGCGAGTACAACCTTCTTG-3′	5′-ATACCCACCATCACACCCTG-3′

Eral1: Era-like 12S mitochondrial rRNA chaperone 1.

**Table 3:** Basic characteristics of Sprague-Dawley rats.

	NDC (*n* = 8)	STZ + NS (*n* = 8)	STZ + SPR (*n* = 8)	*P* values
BW (g)	527.10 ± 8.61	218.90 ± 7.65^∗∗∗∗^	189.10 ± 10.98^∗∗∗∗^	<0.0001
BG (mM)	6.26 ± 0.32	32.69 ± 0.41^∗∗∗∗^	30.50 ± 1.14^∗∗∗∗^	<0.0001
SBP (mm Hg)	136.10 ± 1.82	147.50 ± 5.38	145.50 ± 6.33	>0.05
DBP (mm Hg)	81.13 ± 5.25	81.43 ± 5.39	77.82 ± 3.70	>0.05
HW (g)	1.94 ± 0.09	1.35 ± 0.12^∗∗∗∗^	1.29 ± 0.17^∗∗∗∗^	<0.0001
HW/BW (mg/g)	3.94 ± 0.22	6.68 ± 0.70^∗∗∗∗^	7.22 ± 1.12^∗∗∗∗^	<0.0001

NDC: nondiabetic controls; STZ: streptozotocin; NS: saline; SPR: spironolactone; BW: body weight; BG: blood glucose; SBP: systolic blood pressure; DBP: diastolic blood pressure; HW: heart weight; HW/BW: heart weight/body weight. *N* = 8, data are means ± SEM. ^∗^*P* < 0.05 and ^∗∗∗∗^*P* < 0.0001 vs. NDC group.

## Data Availability

The data that support the findings of this study are available from the corresponding author upon reasonable request.
